# WSSV ie1 promoter is more efficient than CMV promoter to express H5 hemagglutinin from influenza virus in baculovirus as a chicken vaccine

**DOI:** 10.1186/1471-2180-8-238

**Published:** 2008-12-31

**Authors:** Fang He, YuenFern Ho, Li Yu, Jimmy Kwang

**Affiliations:** 1 Animal Health Biotechnology, Temasek Life Sciences Laboratory, National University of Singapore, 1 Research Link, 117604, Singapore; 2 Department of Microbiology, Faculty of Medicine, National University of Singapore, Block MD4, 5 Science Drive 2, 117597, Singapore

## Abstract

**Background:**

The worldwide outbreak of influenza A (H5N1) viruses among poultry species and humans highlighted the need to develop efficacious and safe vaccines based on efficient and scaleable production.

**Results:**

White spot syndrome virus (WSSV) immediate-early promoter one (ie1) was shown to be a stronger promoter for gene expression in insect cells compared with Cytomegalovirus immediate-early (CMV) promoter in luciferase assays. In an attempt to improve expression efficiency, a recombinant baculovirus was constructed expressing hemagglutinin (HA) of H5N1 influenza virus under the control of WSSV ie1 promoter. HA expression in SF9 cells increased significantly with baculovirus under WSSV ie1 promoter, compared with CMV promoter based on HA contents and hemagglutination activity. Further, immunization with baculovirus under WSSV ie1 promoter in chickens elicited higher level anti-HA antibodies compared to CMV promoter, as indicated in hemagglutination inhibition, virus neutralization and enzyme-linked immunosorbent assays. By immunohistochemistry, strong HA antigen expression was observed in different chicken organs with vaccination of WSSV ie1 promoter controlled baculovirus, confirming higher efficiency in HA expression by WSSV ie1 promoter.

**Conclusion:**

The production of H5 HA by baculovirus was enhanced with WSSV ie1 promoter, especially compared with CMV promoter. This contributed to effective elicitation of HA-specific antibody in vaccinated chickens. This study provides an alternative choice for baculovirus based vaccine production.

## Background

The spread of highly pathogenic avian influenza A (H5N1) viruses from Asia to the Middle East, Europe, and Africa poses the threat of an influenza pandemic. Vaccination of poultry is an effective measure to control virus spread [1]. Current production of inactivated influenza vaccine requires high-level biocontainment facilities and large numbers of embryonated chicken eggs, while baculovirus surface displayed recombinant hemagglutinin may be an attractive alternative to the effective influenza vaccine [2-5].

White spot syndrome virus (WSSV), a major pathogen in shrimp, can infect a wide range of invertebrate tissues and cells. WSSV genome has 9 repeated regions similar to those of baculovirus, suggesting the potential to exploit WSSV promoters in baculovirus and insect cell expression system [6,7]. Baculovirus produces high yield of foreign soluble protein in insect cells and mediates efficient transduction of mammalian cells. Thus, it is widely used as a vaccine production system [8]. WSSV ie1 promoter was reported as one of the strongest promoters in insect cells [9,10]. However, no documented report has compared the activity of WSSV ie1 promoter with other promoters in vaccine production. In this study recombinant baculoviruses were constructed under WSSV ie1 promoter, in an attempt to establish a novel platform for efficient antigen expression. These recombinant baculoviruses were further evaluated in the hemagglutinin production of H5N1 influenza virus.

The influenza virus HA glycoprotein has receptor-binding activity and mediates viral-endosomal membrane fusion during viral entry and serves as the primary target for neutralizing antibodies [11,12]. HA protein from H5N1 influenza virus expressed in baculovirus mediated by WSSV ie1 promoter can be displayed on baculovirus surface without disrupting its authentic cleavage, hemagglutination activity and immunogenicity [13]. Besides, baculovirus pseudotyped with the vesicular stomatitis virus glycoprotein (VSV G) emerges as a promising gene-delivery vector by virtue of its capability in transducing numerous mammalian cells [14,15]. Coexpressed with VSV G in baculovirus, the HA protein could be delivered into host cells to elicit immune response in a long term. For the efficient HA delivery to target cells, an active promoter is required in both vertebrate and invertebrate species.

The current study compared WSSV ie1 promoter with CMV promoter in the context of baculovirus vector for the efficient expression of HA protein from H5N1 influenza virus as a surface-displayed immunogen in SF9 (*Spodoptera frugiperda*) cells. Further studies on immunogenicity were performed for these baculovirus vaccines under WSSV ie1 promoter in chickens. The results demonstrated that HA of H5N1 influenza virus could be more efficiently produced by baculovirus with WSSV ie1 promoter, which serves as a safe vaccine in chickens and provides effective immune protection from avian influenza.

## Results

### WSSV ie1 promoter mediates efficient protein expression in SF9 cells

**Figure 1:**
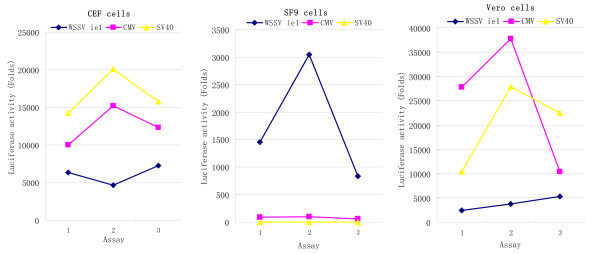
**Comparison of promoter activity of WSSV ie1 and CMV promoter in luciferase assays in different cell lines**. The relative luciferase activity was expressed as fold activity 24 h post-transfection, and the data were from three transfections. The transfections were performed with the reporter plasmid phRL with WSSV ie1, CMV and SV40 promoter individually. CEF: transfections in chicken embryo fibroblasts; SF9: transfections in SF9 cells. Vero: transfections in Vero cells. *p*. value is less than 0.005, when WSSV ie promoter was compared with CMV promoter in SF9 cells, evaluated in *t-test*.

In order to investigate whether the relative strength of the promoter was cell type dependent, a plasmid containing WSSV iel promoter (phRL-ie1) for luciferase expression was transfected into CEF and SF9 cells to test luciferase activity, in comparison to CMV (phRL-CMV). Luciferase activity, indicating intracellular luciferase quantity, was presented in folds of the basic value set in the system. Hence, a link was established between promoter activity and luciferase activity. SV40 promoter was used as a control promoter in both insect and mammalian cells. Vero cells were used to normalize transfection efficiency. CMV promoter activity (mean 87 folds, SD 5.3) was much weaker than the WSSV iel promoter (mean 1610 folds, SD 26.4) in SF9 cells. In CEF cells, the WSSV iel promoter activity (mean 6195 folds, SD 156.8) was slightly less than the CMV (mean 12715 folds, SD 258.8) (Fig 1). The data indicated that the WSSV iel promoter activity was strong in insect cells, in which CMV promoter activity was weak. Furthermore, WSSV ie1 promoter was found to be active in all of the vertebrate cells tested here, including human TK-143b, monkey Marc145, Vero, porcine PK15 and carp epithelioma papillosum (EPC) (data not shown). This property of WSSV ie1 promoter renders it a promising candidate for efficient protein expression in baculovirus infected SF9 cells.

### WSSV ie1 promoter stimulates strong H5 hemagglutinin expression in baculovirus

**Figure 2:**
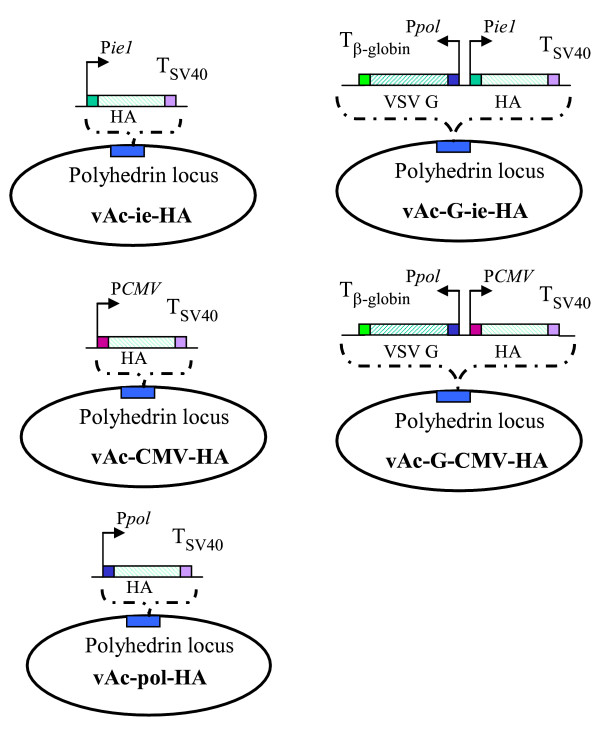
**Schematic representation of the construction of variant baculoviruses in the study**. The HA expression cassettes was inserted under different promoter individually. The desired VSV G expression cassettes was inserted under the polyhedrin promoter. ie: WSSV ie1 promoter; CMV: CMV promoter; pol: Polyhedrin promoter; G: VSV G protein; polyhedrin locus is based on pFast-1 baculovirus vector in Bac-Bac system.

**Figure 3:**
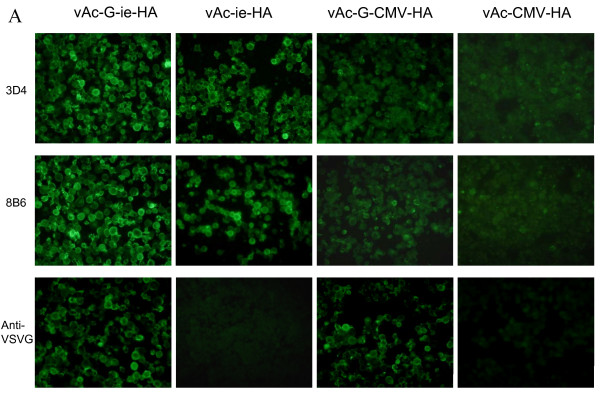
**Efficient production of activated HA protein of influenza virus by WSSV ie1 promoter in baculovirus**. (A) Immunofluorescence assays with different recombinant baculovirus infected SF9 cells. Cells were fixed 3 days post-infection and subjected to antibody staining. 3D4 and 8B6 are H5-specific monoclonal antibodies. Anti-VSVG is anti-VSV G protein antibody. (B) Hemagglutination assays. Every 25 μl of baculovirus at a titer of 10^10 ^PFU/ml was loaded into the standard hemagglutination assay. Data were collected from 4 parallel assays. (C) One-step growth curves of baculoviruses under three different promoters in SF9 cells. Each data point represents the mean value of four individual infections. SF9 cells were infected by individual viruses with a MOI of 0.5 PFU/cell. (D) HA contents of baculoviruses under three different promoters during infection. Data, shown in mean + SD, were collected from 4 parallel assays.

To further compare WSSV ie1 promoter with CMV promoter in the efficiency of protein expression, four recombinant baculoviruses were constructed, termed as vAc-ie-HA and vAc-CMV-HA expressing HA; vAc-G-ie-HA and vAc-G-CMV-HA coexpressing VSV G protein with HA for gene transduction [16,17] (Fig 2). To confirm the activity of WSSV ie1 promoter in SF9 cells as shown in luciferase test, SF9 cells were infected with the four recombinant baculoviruses individually. The infected cells were fixed and subjected to antibody staining 3 days postinfection. 3D4 and 8B6 are two different H5-specific monoclonal antibodies used in these studies [18]. As shown in Fig 3A, indirect fluorescence signals from HA protein were strong and sharp by recombinant baculoviruses with WSSV ie1 promoter. HA expression was detected in cells infected with CMV promoter-controlled baculoviruses though the fluorescence signals were diffused and faded. For those baculoviruses with VSV G cassette, the staining with anti-VSVG antibody verified the successful coexpression of VSV G protein and suggested the selected promoter has no effect on the infection efficiency (Fig. 3A).

Baculovirus-expressed HA sustains hemagglutination activity. The HA titer of baculoviruses under different promoters was evaluated with the same number of virus particles. 25 μl of baculovirus at a titer of 10^10 ^PFU/ml was used in the standard hemagglutination assay. Data were collected from 4 parallel assays. As shown in Figure 3B, constructs under WSSV ie1 promoter gave a higher mean hemagglutination titer of 1:256 (vAc-ie-HA) to 1:320 (vAc-G-ie-HA), while those under CMV were at a mean titer of 1:44 (vAc-CMV-HA) to1:48 (vAc-G-CMV-HA) (*p *< 0.05). Coexpression of VSV G protein had no significant effect on the hemagglutination result (*p *≥ 0.359). Besides, at a mean titer of 1:112, vAc-pol-HA, a HA-expressing construct under baculovirus polyhedrin promoter, was included in this hemagglutination test as a system control, since it is widely used in recombinant baculoviruses for HA production [8]. The HA titer of vAc-ie-HA was higher than vAc-pol-HA at the same virus copies (*p *< 0.0001), indicating its advantage in HA production.

To verify this result, recombinant virus copies and HA contents were measured in a time course study during baculovirus infection. As shown in Fig. 3C, the temporal kinetics of the growth curves for these viruses were similar [13] among the three promoters studied here. However, differences were found in HA production with the three promoters (Fig. 3D). With WSSV ie1 promoter, the HA content in virions was up to 6.6 ug/ml (SD 0.56) corresponding to the virus titer of 10^9 ^PFU/ml at 96h post-infection. The HA content of polyhedrin promoter was 5.05 ug/ml (SD 0.48) at the similar virus titer, while the HA production of CMV promoter was around 2 ug/ml (SD 0.40) at the same collection time. In ANOVA test, *p *values for comparisons among three promoters at each time point were less than 0.005 and the differences between each two groups were considered as significant. (For N tests, p < 0.005 is significant at the overall 0.05 level with Bonferroni adjustment.) Taken together, these results indicated that WSSV ie1 promoter can induce more abundant H5 hemagglutinin expression in baculovirus with hemagglutination activity, in comparison to polyhedrin promoter, as well as CMV promoter.

### Immunogenicity of H5 hemagglutinin expressed by WSSV ie1 promoter in chickens

**Figure 4:**
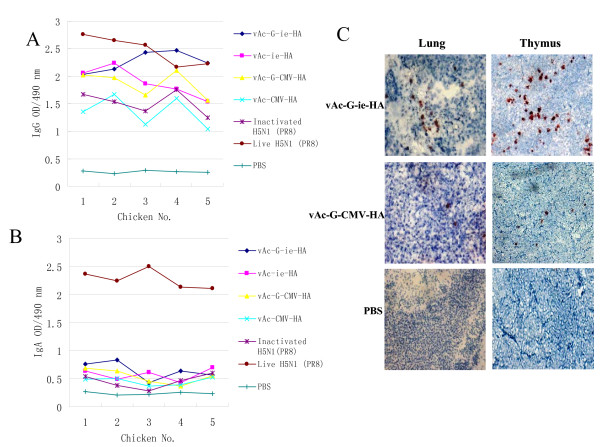
**Immunogenicity of HA-expressing baculoviruses**. (A,) ELISA antibody levels in chicken induced by various immunogens via intramuscular infection. (B) ELISA antibody levels induced by various immunogens via intranasal inoculation. Each group (n = 5) of chickens were immunized with different vaccines at the same dose of virus particles or protein contents. Two weeks after the second vaccination, serum samples from individual chickens in each group were tested by ELISA with purified virion of H5N1 (VNH5N1-PR8/CDC-RG) as antigen. The results are expressed as the absorbance of 1:100 diluted individual sera at OD490. (C) Immunohistochemistry with frozen tissue sections from vaccinated chickens. Thymus was collected from intramuscularly immunized chickens and lung was collected from intranasally immunized chickens. PBS: tissues from control chickens with PBS.

The immunogenicity of baculovirus under WSSV ie1 promoter or CMV promoter was subsequently investigated through intramuscular (IM) or intranasal immunization (IN) of 2-week-old chickens with purified live virions without adjuvant. The live H5N1 vaccine (VNH5N1-PR8/CDC-RG) was used as the positive control, while PBS vaccinated chickens served as negative controls. The same H5N1 (VNH5N1-PR8/CDC-RG) strain was inactivated with BEI (binary ethylenimine) [19] as another control. The serology assays performed here were based on five different serum samples from five individual chickens in the each group (95% confidence interval is between 8.72 and 35.02). To determine the neutralizing antibody level in those chicken sera, HI tests were performed with H5N1 (VNH5N1-PR8/CDC-RG). As shown in Table 1, the WSSV ie1-type baculoviruses elicit higher HI titer than CMV promoter in serum samples. Coexpression of VSV G also contributes to an increase in anti-HA antibody level with HI activity. In addition, to confirm this result about the neutralization activity, a standard micro-neutralization test was performed with H5N1 (VNH5N1-PR8/CDC-RG) in MDCK cells. The sera induced by WSSV ie1-controlled HA-displaying baculoviruses showed a higher neutralization titer than those from CMV promoter, while coexpression of VSV G protein enhanced the neutralization titer in samples of both WSSV ie1 and CMV promoters. Further, serum samples were tested in ELISA. Serum samples from the second collection were diluted at 100 fold in PBS and tested for the anti-HA antibody level. For those samples from intramuscular injection (Fig. 4A), at the same dosage of virus inoculated, the chickens immunized with those baculoviruses under WSSV ie1 promoter developed higher antibody response than those under CMV promoter (*p *< 0.05). Moreover, coexpression of VSV G protein contributed to an increase in anti-H5 antibody level (*p *< 0.05) due to the transduction mediated by VSV G protein. In the lack of constant virus replication *in vivo*, antibody levels of these WSSV ie1 baculovirus immunized chickens were relatively lower than those of live H5N1 (VNH5N1-PR8/CDC-RG) infected chickens (*p *≤ 0.09), but they were higher than those of immunized chickens with inactivated H5N1 (VNH5N1-PR8/CDC-RG) at the same protein dosage (*p *< 0.05). For those intranasally immunized chickens by baculovirus, lower IgG response was detected compared with intramuscularly injected chickens (Fig. 4B). Furthermore, cut-off value of this ELISA was determined as 0.3 based on tests with healthy new-born chicken serum. To further evaluate the HA-specific antibody level in sera, the dilution factor of each serum sample was recorded (Table 1) at the value beyond 0.3 in ELISA. The data obtained in this method is consistent to the results from other tests performed here. All of these findings indicated that efficient production of HA by WSSV ie1 promoter in baculovirus allowed it to be exploited as a vaccine production platform.

**Table 1:** Elicitation of influenza A virus HA specific antibody in chickens immunized with HA expressing recombinant baculovirus.

Inoculum	Dose	Route	HI GMT (range)		VN GMT (range)		ELISA GMT (range)	
			
			1st Dose	2nd Dose	1st Dose	2nd Dose	1st Dose	2nd Dose
vAc-G-ie-HA	10^9^ PFU	IM	6.4 (4–8)	204.8a (128–256)	32 (20–40)	192a (160–320)	384 (320–640)	12288a (10240–20480)

vAc-ie-HA	10^9^ PFU	IM	3.2 (2–4)	11.2b (8–16)	8 (<10–10)	28b (20–40)	224 (160–320)	5120b (5120)

vAc-G-CMV-HA	10^9^ PFU	IM	2.4 (2–4)	76.8c (64–128)	16 (10–20)	112c (80–160)	288 (160–320)	9216c (5120–10240)

vAc-CMV-HA	10^9^ PFU	IM	1.6 (<2–2)	4.8d (4–8)	6 (<10–10)	13d (<10–20)	192 (160–320)	2560d (2560)

H5N1/PR8 Inactivated	6 ug	IM	2 (2)	5.6 (4–8)	7 (<10–10)	22 (<10–40)	64 (40–80)	3072 (2560–5120)

H5N1/PR8	6 ug or 10^5^ TCID50	IM	96 (32–128)	1024 (1024)	64 (40–80)	352 (160–640)	2560 (2560)	>20480 (20480)

PBS	100 ul	IM	1 (<2)	1 (<2)	5 (<10)	5 (<10)	5 (<10)	5 (<10)

HI: hemagglutination inhibition titer of serum from immunized chickens. VN: virus microneutralization titer of serum from immunized chickens. GMT: geometric mean titer. GMT is calculated based on 5 different sera from 5 different chickens in each groups. For titers <2 in HI, 1 was used to calculate the GMT. A GMT of 1 was considered negative. For titer <10 in VN and ELISA, 5 was used to calculate the GMT. A GMT of 5 was considered negative. 1^st ^Dose: serum samples collected at 2 weeks from the first inoculation. 2^nd ^Dose: serum samples collected at 2 weeks from the second inoculation. IM: intramuscular.

^a-d ^*p *< 0.05. Values denoted by different letters are statistically different from each other in the same comparison, as analyzed in ANOVA test (not adjusted).

### Significant antigen expression in chicken tissue was induced by HA-VSV G coexpression constructs

To further study HA-transduction in immunized chickens and detect antigen expression in tissue based on vaccination, immunohistochemistry of frozen tissue sections from chickens was performed with an H5-specfic monoclonal antibody 2 weeks after the second vaccination. Four chickens of each group were tested. Similar results were found among 3 chickens in the group of vAc-G-ie-HA by IM, 4 of vAc-G-ie-HA by IN, 3 of vAc-G-CMV-HA by IM and 2 of vAc-G-CMV-HA by IN. In both of the two promoter groups, signals were mainly observed in the lung tissues (intranasal inoculation) and thymus tissues (intramuscular inoculation). As shown in Fig 4C, in frozen tissue sections from chickens inoculated with recombinant HA-expressing baculovirus under WSSV ie1 promoter (vAc-G-ie-HA), signals of HA expression appeared intensive and distributed densely. In tissues from chickens inoculated with recombinant baculovirus vAc-G-CMV-HA, sporadic signals for HA expression were observed. These results gave direct evidence of successful protein transduction by VSV G-expressing baculoviruses. In addition, the recombinant baculovirus under WSSV ie1 promoter was shown to bring strong antigen expression in chicken tissues, which depends on its activity of HA expression in both of insect and chicken cells.

## Discussion

The recombinant baculoviruses under WSSV ie1 promoter presented here, present advantages in HA production and gene transduction, relying on its promoter efficiency in both vertebrate and invertebrate species. In this study, CMV promoter was used for major comparison because it displayed activity in both mammalian and insect cells as indicated in luciferase assays and it is widely used for protein expression and gene transduction in numerous cell lines [20-22]. However, CMV promoter might be relatively weak for protein expression in insect cells. Therefore, WSSV ie1 promoter was also compared with polyhedrin promoter from baculovirus in HA expression in insect cells. The results confirmed the role of WSSV ie1 promoter as an efficient promoter for baculovirus mediated protein expression. HA expressed in baculovirus served as exogenous antigen to stimulate primary immune response, while HA *de novo *expression in chicken tissue will contribute to the trigger of new HA-antibody production for further protection in a long run [18,20]. For gene transduction, although CMV promoter is stronger in chicken cells than WSSV ie1 promoter, as indicated in luciferase assays, such advantage could be offset by the stronger HA expression on viral surface with WSSV ie1 promoter, which eventually leads to enhanced HA-specific immune response in chickens [23].

To further verify those advantages brought by WSSV ie1 promoter in vaccine production, the immunogenicity of these baculovirus-based immunogens was studied with chickens. In the comparison of different promoters in the same type of baculovirus construct, vaccine dose was based on virus copies rather than protein contents in order to differentiate the HA production efficiency by different promoters with the same amount of baculovirus copies (10^9 ^PFU) [8]. As shown here, at the same dosage of baculovirus, constructs with WSSV ie1 promoter elicited better immune response than CMV promoter, confirming the higher HA expression level by WSSV ie1 promoter. Meanwhile, when the comparison was performed between baculovirus and attenuated H5N1 influenza virus, dosage was based on HA contents (6 ug) due to differences in viral property.

As the direct evidence for gene transduction, HA antigen expression in tissues was revealed in IHC assays. The results were repeatedly observed in most chickens from each group and considered to be significant. For intranasal inoculation, lung is the major organ to directly contact immunogen upon vaccination, where VSV G mediates virus entry and HA expresses with its individual promoter. Also, as the major organ involved in immunity of avian species, thymus supports exogenous antigen expression [24-26]. Therefore, for intramuscular injections, most of HA expression was detected in chicken thymus.

Although baculovirus-expressed hemagglutinin influenza vaccines have been widely used and well characterized in different ways and under variant vector designs [5,8,27-29], innovative methods are under investigation for more efficient HA production at a higher hemagglutination titer. WSSV ie1 promoter supports the abundant production of HA in baculovirus system, as compared with other promoters tested here. This allows it to induce higher level of specific antibody response in immunized poultries at the same number of baculovirus copies. In addition, compared with inactivated H5 influenza virus vaccines at the same dose of HA protein (*p *< 0.05), data shown here indicates that WSSV ie1 promoter-mediated baculovirus vaccine could present better immunogenicity without biosafety concerns in vaccine preparation [30]. This also suggests that there could be some other properties of WSSV ie1-controlled baculovirus contributing to better immunogenicity. One possibility is that the surface-displayed HA in baculovirus sustains its natural conformation upon vaccination due to the obviation of the inactivation process in the baculovirus-type vaccine production [8]. Future studies will focus to identify whether other properties of WSSV ie1 promoter support strong immunogenicity. Taken together, our studies provide an alternative choice for the efficient production of surface-displayed HA with baculovirus. Its vaccine potential was primarily studied in chickens, which might throw light on its promising trials in humans.

## Conclusion

With WSSV ie1 promoter, the recombinant baculovirus provided an efficient and expeditious method in vaccine production, compared with traditional means. WSSV ie1 promoter-mediated baculovirus conferred better immunogenicity in chickens upon vaccination in the light of the efficient HA expression in both insect and chicken cells. This study fully characterized the capacity of baculovirus featuring WSSV ie1 promoter in antigen production and immune response elicitation in chickens, suggesting it could be a promising choice as an efficient vaccine production system.

## Methods

### Viruses and cells

VNH5N1-PR8/CDC-RG obtained from Center for Disease Control (USA) is a non-pathogenic H5N1 influenza virus. PR8 strained-based reassortant virus comprises of the HA and NA gene of AIV H5N1 virus infecting human in Vietnam (A/Vietnam/1203/04). The virus was grown in the allantoic cavities of 10-day-old embryonated eggs (Chew's Poultry Farm, Singapore).

Madin-Darby canine kidney (MDCK) cells and African green monkey cell line (Vero) were obtained from American Type Culture Collection (ATCC) and grown in DMEM containing 10% FBS at 37°C with 5% CO_2_. *Spodoptera frugiperda *pupal ovarian (SF9) cells (Invitrogen) were grown at 28°C in serum-free medium Sf-900 II SFM (GIBCO BRL) supplemented with 100 μg/ml gentamycin and transfected with Effectene transfection reagent (Qiagen). Primary chicken embryo fibroblasts (CEF, prepared from specific-pathogen-free chicken embryo, Chew's Poultry Farm, Singapore) were cultured in RPMI 1640 (GIBCO BRL) medium supplemented with 10% fetal bovine serum and 100 μg/ml penicillin and 100 μg/ml streptomycin at 37°C under a 5% CO_2 _atmosphere.

### Luciferase activity assay

Renilla luciferase activity was measured with the Dual-Luciferase Reporter Assay System (Promega, Madison, WI) according to the protocol (Technical Manual, #TM040) using a Luminometer (Berthold, Lumat LB 9507, ITS Science & Medical PTE LTD) [31]. DNA was isolated from WSSV-infected shrimps using DNeasy tissue kit (Qiagen). Luciferase reporter plasmids were constructed by inserting WSSV ie1 promoter into KpnI-Hind III sites of phRL vector. Vector pRL-SV40 and pRL-CMV were provided in the kit. Cells were lysed in 1 × lysis buffer (50 μl/well) for 15 min at room temperature and each cell lysate was added into the luminometer tube containing 100 μl of assay reagent. The mixture was mixed quickly by flicking for 2 s, and placed in the luminometer for 10 s measurement. Transfection efficiency was normalized using pRL-SV40 in Vero cells, in which SV40 promoter drives the firefly luciferase reporter gene. Data (mean + SD) were collected from triplicate assays of three independent transfections.

### Construction of recombinant baculoviruses

For the generation of the recombinant baculovirus vectors, as mentioned before [13], AcMNPV polyhedrin promoter-controlled vesicular stomatitis virus glycoprotein (VSV G) [17] expression cassette and WSSV ie1 promoter or CMV promoter-controlled HA expression cassette (Figure 1) were inserted into the shuttle vector pFastBac1 and integrated into the baculovirus genome within DH10BAC™ according to the protocol of Bac-To-Bac system (Invitrogen). HA gene in our study was amplified from a Vietnam strain (A/Vietnam/1203/04/H5N1) with the multibasic HA cleavage site in a standard PCR method (94°C 20 sec, 55°C 30 sec and 72°C 2 min for 30 cycles). CMV-HA cassette was amplified with a primer set of 5' ACGCTACGTATAGTTATTAATAGTAATCAA 3' and 5'ACGTGCGGCCGCTTAAATGCAAATTCTGCATTGTAACGATC3' from vector pCMV-EGFP (BD clontech) with HA gene. CMV promoter in the vector is Human cytomegalovirus (CMV) immediate early promoter without intron. The CMV-HA cassette was inserted into pFastBac1 at the SnabI-NotI site. HA was also inserted into pFastBac1 vector with polyhedrin promoter at the NotI-SalI site to generate vAc-pol-HA recombinant baculovirus.

### Recombinant baculovirus purification

Infected SF9 cells with individual recombinant baculoviruses were harvested at 96 h postinfection and subjected to freeze-thaw cycles for cell lysis. The cell lysate was spinned at 1000 g for 5 minutes to remove cell debris. From the supernatant, the virus was purified by sucrose gradient ultracentrifugation following standard protocols [8] and the purity was determined by SDS-PAGE. Virus titer was determined in standard plaque assays with SF9 cells according to baculovirus construction protocol (Invitrogen, No.10359). Hemagglutinin contents in purified virions were estimated by densitometric analysis of stained gels following electrophoresis with Quantity One software (Bio-Rad).

All of the baculoviruses were used without inactivation. As a control of protein vaccine, H5N1 (VNH5N1-PR8/CDC-RG) strain was inactivated with 0.3 M BEI (binary ethylenimine) incubated at 37°C overnight, according to the previous protocol [19].

### Animal experiments

14-day-old chickens (Chew's Poultry Farm, Singapore) received two doses of vaccines or PBS at intervals of 14 days by intramuscular injection or intranasal inoculation. 5 chickens were in each group. Each chicken was vaccinated with purified live baculoviruses without adjuvant at the dose of 10^9 ^PFU based on virus copies (100 ul, 10^10 ^PFU/ml), or with influenza virus without adjuvant at the dose of 6 ug based on HA content or 10^5 ^TCID_50 _based on infectivity. The sera were collected two weeks after each vaccination for evaluation. Chickens were killed for dissection two weeks after the second vaccination.

Approval for the animal experiments was obtained from Institutional Animal Care and Use Committee in Temasek Life Sciences Laboratory, Singapore (the approved project number TLL-07-007).

### Serological assays

To inactivate non-specific inhibitors, chicken sera were treated with receptor destroying enzyme (RDE, Sigma) by incubation at 56°C for 30 min. Hemagglutination inhibition (HI) tests were carried out in microtitre plates with 1% suspension of chicken red blood cells. For neutralization tests, 2 × 10^4^/ml of MDCK cells were allowed to grow to 70% – 90% of confluence. Allantoic fluids with H5N1(VNH5N1-PR8/CDC-RG), using a series of dilutions factors from 10^-1 ^to10^-8^, were tested for TCID_50_. Using Reed and Muench mathematical technique., the infectivity titer was expressed as TCID_50_/100 μl and the viruses (VNH5N1-PR8/CDC-RG) were diluted to having 100 TCID_50 _in 50 μl. After which, 100 TCID_50_viruses were incubated with chicken serum for 1 h at 37°C and inoculated into MDCK cells. The cells were then incubated at 37°C and CPE was observed at 96 h post-infection. Besides, a cell-based ELISA were performed to determine neutralization titer according to standard procedures [32,33]. Guinea pigs were immunized with a live non-pathogenic virus (VNH5N1-PR8/CDC-RG) at 6 ug of HA contents and bled after two injections. The guinea pig IgG was purified from serum using protein A affinity column (Sigma, USA) in accordance with manufacturer's instructions. Enzyme-linked immunosorbent assays (ELISA) were performed by antigen-capture methods with purified anti-H5N1 IgG from guinea pig (150 ng/well) as capturing antibody. The plate was then incubated with purified virus (VNH5N1-PR8/CDC-RG) as antigen. The chicken serum samples were added and anti-chicken IgG secondary antibody HRP (Sigma, 1:3000) was used to develop signals with OPD substrate (Sigma, 1 tablet in 20 ml water).

### Immunofluorescence assays

SF9 cells were infected with HA-expressing baculovirus. They were fixed at 3 days post-infection with 100 μl of absolute ethanol for 10 minutes at room temperature. Cells in 96-well plates were then washed 3 times with PBS, pH 7.4. Subsequently, the fixed cells were incubated with 50 μl of anti-H5 [18] or anti-VSVG monoclonal antibody (Sigma) for 1 hr at 37°C. After 3 washings, the antigens were incubated with fluorescein isothiocyanate (FITC) – conjugated anti-mouse Ig (1:100 DAKO, Denmark) for 1 h at 37°C. The cells were observed under fluorescence microscope.

### Immunohistochemistry

Chickens were dissected after 2 weeks from the second vaccination and a series of organ tissues were collected, including brain, kidney, liver, lung and spleen. They were in the form of frozen sections. Commercially available immunoperoxidase staining system (Dako Cytomation EnVision + System-HRP (AEC)) was used for these specimens according to instructions in the kit. This is a two-step staining technique. to recognize bound antibodies. based on a horseradish peroxidase labeled polymer which is conjugated with secondary antibodies.

### Statistical analysis

Welch's t test, which is the two-sample t-test that does not assume equal variances between groups, was performed to determine the level of significance in the difference between means of two groups (GraphPad, Software). One way ANOVA was performed by using ANOVA test calculator (Danielsoper, Software) online and the level of significance of difference in multiple comparison was determined according to Bonferroni adjustment (α = 0.05) for multiple comparisons if applicable. 95% confidence interval was determined using survey calculator online (Creative research systems).

## Authors' contributions

FH carried out the experiments, analyzed the data and drafted the manuscript, YH performed animal vaccination and immunohistochemistry, LY performed luciferase studies and JK contributed to the experimental design of the study and critical analysis of the data.
